# Utilizing online resources for taxonomy: a cybercatalog of Afrotropical apiocerid flies (Insecta: Diptera: Apioceridae)

**DOI:** 10.3897/BDJ.3.e5707

**Published:** 2015-10-06

**Authors:** Torsten Dikow, Donat Agosti

**Affiliations:** ‡ National Museum of Natural History, Smithsonian Institution, Washington, DC, United States of America; § www.plazi.org, Bern, Switzerland

**Keywords:** cybertaxonomy, open-access, online repositories

## Abstract

A cybercatalog to the Apioceridae (apiocerid flies) of the Afrotropical Region is provided. Each taxon entry includes links to open-access, online repositories such as ZooBank, BHL/BioStor/BLR, Plazi, GBIF, Morphbank, EoL, and a research web-site to access taxonomic information, digitized literature, morphological descriptions, specimen occurrence data, and images. Cybercatalogs as the one presented here will need to become the future of taxonomic catalogs taking advantage of the growing number of online repositories, linked data, and be easily updatable. Comments on the deposition of the holotype of *Apiocera braunsi* Melander, 1907 are made.

## Introduction

Cybertaxonomic tools enable us to utilize web-based databases and data repositories to store and retrieve information on taxon names, publications, digitized literature, morphological descriptions, molecular sequences, occurrence data, or images. The availability of these kinds of data in an open-access, online framework allows scientists to test and support taxonomic and phylogenetic hypotheses readily​ as well as link data in support of biodiversity research across taxon boundaries. Furthermore, future research programs are enhanced by re-using and re-purposing available data in analyses and syntheses. The open-access movement encourages researchers to share primary data (Costello 2009), the number of digital repositories is only rising, and data journals can make sharing exceedingly easy (Smith et al. 2013).

There is currently no central online gateway to deposit all of the above kinds of data. However, the Encyclopedia of Life (EoL) strives to present a species page for each known taxon summarizing diverse data from disparate online sources. As a data aggregator, it relies on information being either stored elsewhere and harvested regularly or entered directly into the EoL database.

The present cybercatalog attempts to summarize available information on Afrotropical Apioceridae and provide unique identifiers or URLs to access these data online. While the EoL species page will provide several of the data kinds, it lacks others such as ZooBank unique identifiers. Furthermore, only through the upload of images of Afrotropical *Apiocera* species to Morphbank or taxonomic treatments to Plazi by the authors, for example, EoL is in a position to harvest and include these data in the respective species page.

The hope is that this cybercatalog will encourage entomologists to utilize available cybertaxonomic tools in their research and publications and upload previously or newly published information to databases and data repositories (Costello 2009​) to allow aggregators such as the EoL to make them more widely accessible beyond the primary users such as the taxonomic community.

### Definition of cybercatalog

The term "cybercatalog" is here used to denote a taxonomic catalog, which is referring the reader to taxon-specific, open-access information on the world wide web. The name should not be confused with web-sites that provide access to numerous mail-order catalogs as revealed by searching the world wide web.

### Goal of this cybercatalog in the context of the *Manual of Afrotropical Diptera*

The forthcoming *Manual of Afrotropical Diptera* (*MAD*, web-site) edited by Ashley Kirk-Spriggs and Bradley Sinclair will be an outstanding resource on the status of research on Diptera occurring in the Afrotropical Region. The taxon-specific chapters will address each of the 109 families known to occur in this zoogeographic region and provide identification keys to the genera and a synopsis of each genus. The manual will cover the Afrotropical Region as proposed by Crosskey and White (1977) and Crosskey (1980) plus the Arabian Peninsula countries of Oman and the United Arab Emirates.

The last comprehensive catalog to the genera and species of Afrotropical flies was published 35 years ago by Crosskey (1980). Since then, numerous new genera or species have been described and taxonomic changes published (in Apioceridae, for example, Yeates 1994). While it is understandable that the *Manual of Afrotropical Diptera* cannot serve as an updated catalog to genera and species, which is outside of the scope of the project, there is an opportunity to publish taxonomic catalogs in open-access electronic format as data papers prior to (or almost simultaneous with) the publication of *MAD* volumes. Because *MAD* will be published as a traditional book and the inclusion of unique identifiers (such as Globally Unique Identifiers GUIDs or Life Science Identifiers LSIDs) and URLs to data repositories harboring a wealth of information will not be possible, data papers can be cited and the reader will have access to more detailed information on taxa that cannot be included in the general taxon-specific chapters in the *MAD*.

While the species diversity of Apioceridae in the Afrotropical Region is small, currently only three species are known, this catalog should be seen as an example highlighting the usefulness of how online, open-access resources can provide valuable taxon and specimen information. Furthermore, it highlights the need to organize research information and unique identifiers locally (see below) in order to export them for inclusion in data papers. The *BDJ Checklist* template, which has been employed here, allows for the easy import of a taxonomic catalog for publication as well as an update of a previously published catalog.

### Future updates to cybercatalogs and data papers

The present cybercatalog includes a novel feature available in the *Biodiversity Data Journal* (BDJ) publishing platform in that this catalog can be updated easily in the future and re-published under a new Digital Object Identifier (DOI) should a new species be described or other taxonomic changes be made. This feature will facilitate the continuous update of the taxonomic information not possible through traditional publishing means in book or article form.

## Materials and methods

The LSIDs (Life Science Identifier), GUIDs (Globally Unique Identifier), UUIDs (Universally Unique Identifier), and URLs (Uniform Resource Locator) to access data in the various repositories listed below are locally stored in a custom FileMaker Pro database (Fig. 1​). This simple taxonomic database has been developed by the senior author to capture information on taxa for research purposes. Over the years, it became evident that the growing number of web addresses providing access to online information on taxa needed to be stored in an easily retrievable way. Therefore, fields were added that either store the unique identifiers (*e.g.*, ZooBank) or web addresses (*e.g.*, Encyclopedia of Life) to data repositories.

**Figure 2.**
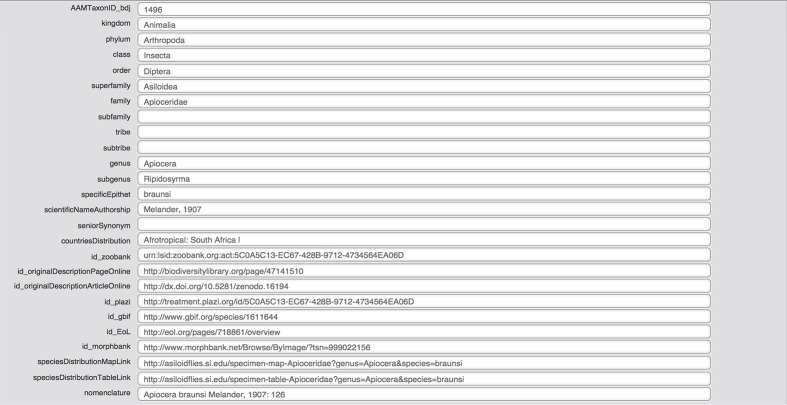
Screenshot of the FileMaker Pro taxon database showing BDJ taxon export layout.

A custom layout (Fig. 2) has been added to export a subset of data according to the *Biodiversity Data Journal Checklist* template. The resulting Excel file needs minor adjustments to comply with the column names in the template before upload into the Pensoft ARPHA Writing Tool can be achieved.

Of the unique identifiers provided below, only the ZooBank and Plazi GUIDs, consisting of a 32 alpha-numeric UUID, are Globally Unique Identifiers (GUID) whereas all other identifiers are unique within their respective data storage system. However, they should not be seen as less permanent because the identifiers will provide a permanent and persistent way to link to the resource (as long as funding of the data storage system is continued). GUIDs, Digital Object Identifiers (DOIs) or Archival Resource Keys (ARKs), would be preferable (Page 2008​, Guralnick et al. 2015​), but minting these identifiers is not supported by all repositories (yet). The ZooBank and Plazi GUIDs listed below will in the future be identical as Plazi now derives treatment GUIDs (see below) from the respective ZooBank taxon registration record.

## Data resources

The data repositories included in this cybercatalog are listed below together with introductory information. While this sort of detail is obviously not needed in a taxonomic catalog, it is included here to make the reader familiar with different tools and in order to encourage the taxonomic community to utilize these repositories in research and publications.

### ZooBank

ZooBank (Pyle and Michel 2008) is a registry of nomenclatural acts, published works, and authors and currently supports only the registration of new taxa (genus- and species-group), but will in the future also be able to accept new synonymies and new combinations, for example. As such it is the *de facto* place where one should be able to find detailed information on each genus- and species-group name.

The present catalog includes the ZooBank GUID to all taxa, which have been registered for the purpose of this catalog. For each nomenclatorial act (*e.g.*, new taxon name) Zoobank issues a UUID as part of the LSID, which is minted. Zoobank and Plazi (see below) share the UUID allowing the retrieval of the related treatment (see below) by adding the respective treatment-specific prefix.

ZooBank will also be the *de facto* resource to resolve authors including all of her/his publications containing new taxon descriptions, *e.g.*, GUID for T. Dikow = F8869067-4618-4CCE-960C-E8A107F162FB. It can also serve as a summary of all nomenclatural acts, such as a count of newly described species by an author, by utilizing the ZooBank API.

The ZooBank records provide access to:

- a genus-group name: the record will show the original work in which the name was established along with the page number and type species. If the original work is available in digital format through, for example, the Biodiversity Heritage Library (see below) it can be directly accessed.
- a species-group name: the record will show the original work in which the name was established along with the page number and any figures in which the species is depicted. Furthermore, information on the number and depository of the primary and secondary type specimens as well as the type locality with geographic co-ordinates, if available, are included. Should the original publication be available online, it can be directly accessed.

### Biodiversity Heritage Library, BioStor, and Biodiversity Literature Repository

The Biodiversity Heritage Library (BHL) is a digital archive of natural history literature and works collaboratively to make biodiversity literature openly available as part of a global biodiversity community. It digitizes any natural history literature that is out of copyright and published prior to 1923. In copyright books and journals can also be digitized by BHL after an agreement with a publisher has been signed. The community can propose titles to be digitized by BHL free of charge by entering information on the BHL scanning request form.

BioStor (Page 2013) works with BHL content and itemizes the digitized journal volume into the respective articles, which are the standard unit of citation. It also makes the itemized article available as a single page PDF download and therefore provides a great resource to obtain open-access publications digitized by the BHL. (Note that BioStor used to provide full-text PDFs of the entire itemized article and this function might be enabled again in the future (R. Page pers. comm.).) BHL has its own itemization and download tool ("Select pages to download" under the "Download Contents" section) and in the past stored such PDFs in CiteBank and provided a unique URL to the article PDFs. CiteBank and these URLs are not supported anymore, but the previously itemized articles should still be available on the main BHL search interface.

The Biodiversity Literature Repository (BLR) is part of CERN’s digital Zenodo archive for scientific data. The BLR is focused on biodiversity literature, specifically articles and illustrations. The items stored are at article- or subarticle-level (*e.g.*, individual treatments) for which a DataCite Digital Object Identifier (DOI) is provided, which allows for a citation of the item in a standardized way. Furthermore, it has the potential to assign DOIs to all legacy literature and with that make these articles first-class citizens. BLR is currently administered by Plazi (see further information below) and Pensoft. Upload of articles is open and free to anybody. Generally, any article published before 2000 without a DOI can be made accessible. BLR is mainly focused on providing access to collections covering specific taxa, regions or subjects such as all ant, proctotrupine or drosophilid taxonomy.

The DOIs/URLs provide access to, if applicable:

- the BHL page on which a particular taxonomic name was established to read the original description and obtain other information (labeled "**original description page online**")
- a variety of digital archives, for example, BHL (first page of an article not yet itemized by BioStor), BioStor (article single page PDFs), BLR (article PDF), publisher (article PDF), or other digitization archive (*e.g.*, Sabinet African Journal Archive (AJA), article PDF) (labeled "**original article online**" **+ "BioStor/BLR/Publisher/AJA"**).

### Plazi

Plazi is an association supporting and promoting the development and service of persistent and openly accessible digital taxonomic literature and its contents. The main emphasis is to provide human- and machine-readable access to taxonomic treatments and data therein as well as to make them easily citable and retrievable. A treatment is a part of an article that is explicitly provided by an author to define his understanding of a taxonomic name usage at the time of publication (Catapano 2010). Treatments can either be provided for new species or for re-descriptions and include references to previous name usages and treatments. Specifically minted persistent URI-based identifiers for treatments allow treatments to be cited analogous to publications (see, *e.g.*, treatment for *Apiocera alastor* (Walker, 1949) by Yeates 1994). In the future, the UUID part of the identifier will be synchronized with ZooBank for the same taxonomic name usage, whereby ZooBank provides facts of the names and Plazi the respective treatment.

Through the use of the GoldenGATE editor, taxonomic articles can be marked-up in XML making the underlying information (such as taxonomic names, descriptions, diagnoses, etymology, material examined, type locality, and notes) accessible in machine-readable form for harvest by aggregators. Both, previously (retroactive) or newly (proactive) published articles/books can be marked-up to extract the species descriptions (Miller et al. 2012) and the resulting treatment pages, *e.g.*, the Mydidae *Namibimydas psamminos* Dikow, 2012 (Dikow 2012), summarize all information originally published on the species. Future studies on this species, such as a new revision including additional specimens or characters in light of new knowledge, should also be made available in Plazi so that taxonomists world-wide will have online access to the original species descriptions and all subsequent re-descriptions.

While the text of the original species description might be available through the BHL, BioStor, or BLR portals, Plazi provides a machine-readable version in TaxonX mark-up language and Resource Description Framework (RDF) that focuses on easy and consistent retrieval of content so that comparison of multiple descriptions dealing with the same or different taxa can be achieved. Plazi treatments can also be used for quantitative comparative studies of published taxonomic research (Miller et al. 2015​).

In collaboration with Pensoft and the U.S. National Library of Medicine, a taxonomy-specific Journal Article Tag Suite (JATS), called TaxPub, has been developed that includes treatments and other elements specific to taxonomic publications and which is now underlying Pensoft journals such as *Biodiversity Data Journal* or *ZooKeys* (Penev et al. 2012). This workflow allows to create machine-readable, semantically-enhanced taxonomic articles during the publishing process. Treatments and more detailed facts are tagged, which facilitates automatic export of treatments and included structured data such as occurrence/observation records to aggregators like EoL (see below) and GBIF, respectively.

The Plazi persistent identifiers/queries provide access to:

- the original description treatment (labeled "**Plazi**")
- all treatments (*e.g.*, original description plus every subsequent re-description) of a given taxon (labeled "**Plazi taxon query**").

### Global Biodiversity Information Facility

The Global Biodiversity Information Facility (GBIF) is a data aggregator that gathers specimen occurrence data from numerous natural history collections and herbaria around the world. It provides free and open access to biodiversity data on all types of life on Earth.

An increasing number of entomological collections have started to digitize their holdings by databasing the specimen occurrence data and submitting them to GBIF. Once the collecting localities have been geo-referenced, GBIF will include the specimens in global distribution maps. Data can also be directly submitted by a researcher or a natural history collection to GBIF through the use of an Integrated Publishing Toolkit (IPT Robertson et al. 2014). For example, Dikow and Leon (2014) submitted the specimen data resulting from a revision of the Mydidae genus *Namadytes* Hesse, 1969, which originated from ten different natural history collections, as GBIF resource 5e6acf4c-e913-45fd-8466-5c0b92c322dd (DOI 10.3897/bdj.2.e1071) using an IPT instance. A third way to upload data to GBIF is through articles in which occurrence/observation records are either marked-up *a priori* (*e.g.*, Biodiversity Data Journal) or *a posteriorly* (Plazi treatments) and made accessible via a Darwin-Core Archive (DwC-A) to GBIF. Through these institutional, personal, or publication-based uploads to GBIF the global distribution maps are enhanced regularly and will provide a more complete picture of the distributional range of taxa based on explicit specimen data.

The GBIF URLs provide access to:

- the respective taxon page with the global distribution.

### Morphbank

Morphbank :: Biological Imaging is an image repository for scientific images of organisms and parts thereof. It provides permanent, open-access to the "published" images and the user can access the original image files. This, for example, allows users to zoom in to see more detail than is available when an image is published in a traditional book or journal article. Furthermore, the user can access specimen-level information, *e.g.* collecting locality, unique specimen identifier, and institutional depository of the photographed specimen as well as imaging technique, and specifics of the view presented. When submitting images to Morphbank, the user has the option to allow the images to be harvested by the EoL (see below) and so the same image can be available on both the Morphbank platform and on the EoL species page. However, a separate entry to Morphbank is advantageous here as not every user provides access to their images on the EoL. Since the number of images of insect specimens from museum collections and publications of a particular taxon will only increase, Morphbank can be an ever-increasing resource for digital photographs.

The Morphbank URLs provide access to:

- the respective taxon page summarizing all images available under that specific name (labeled "**Morphbank (by taxon)**").

### Encyclopedia of Life

The Encyclopedia of Life (EoL) is a data aggregator that harvests information such as scientific names, images, descriptions, digitized literature, and others pertaining to species or higher taxa. It attempts to provide a summary page, the so called species page, for every single species and higher taxon known to science and provides all information in an open-access framework.

Some of the data provided on the EoL species page is duplicated from other individual data sources employed here, such as GBIF and Morphbank. Similar to GBIF, the EoL can also directly receive data, such as images, from museum collection databases or treatments from Pensoft journals, *e.g.*, *ZooKeys*, or Plazi. For example, images uploaded into the Smithsonian Institution's National Museum of Natural History (USNM) KE EMu specimen-level database will be shown on the EoL species page (compare images of the USNM record of the holotype of *Apiocera braunsi* to the EoL species page for that taxon).

The EoL URLs provide access to:

- the respective taxon page.

### Research web-site asiloidflies.si.edu

The research web-site of the senior author (asiloidflies.si.edu) provides access to various data on Apioceridae, Asilidae, and Mydidae flies using the Drupal content management system.

The URLs provided will take the user directly to either an interactive distribution map or a table with specimen occurrence data of the respective taxon. These specimen records were gathered and geo-referenced by the senior author from numerous natural history collections. The records will in part be duplicating results shown in GBIF, but will also include records not included in GBIF because so far only a limited number of institutions provide geo-referenced specimen-level records to GBIF and the vast majority of entomological collections have not been digitized at the specimen level. Through the senior author's continued efforts in databasing specimens of Apioceridae and related taxa for research purposes, the distribution maps are enhanced regularly and will provide a more complete picture of the ranges of taxa based on explicit specimen data. For convenience, the same specimen records can also be accessed in table format to more easily search the data.

The research web-site URLs provide access to:

- a distribution map (labeled "**asiloidflies.si.edu distribution map**")
- a table (labeled "**asiloidflies.si.edu specimen table**")

### Institutions holding specimens

To facilitate access to specimens for further research, the notes section includes a list of institutions that have specimens of the respective species in their holdings. It should be noted that additional museum collections might have specimens available as this list is based on visits by the senior author to numerous institutions including those holding the majority of Afrotropical Diptera. A link to the record in the Global Registry of Biodiversity Repositories (GRBio) of that institution is provided to disambiguate the institutional acronym.

## Checklists

### Cybercatalog to genus- and species-group names

#### Apiocera

Westwood, 1835

urn:lsid:zoobank.org:act:B653DD23-4F34-4E6A-90EF-3A781066A923

http://biodiversitylibrary.org/page/2548917

http://dx.doi.org/10.1080/14786443508648642

http://biodiversitylibrary.org/page/2548916

http://treatment.plazi.org/id/DFE567BB-19D7-90B4-F081-A45EF7A98A33

http://plazi.cs.umb.edu/GgServer/search?taxonomicName.isNomenclature=true&taxonomicName.exactMatch=true&taxonomicName.genus=Apiocera

http://www.gbif.org/species/1611607

http://www.morphbank.net/Browse/ByImage/?tsn=132326

http://eol.org/pages/55333/overview

http://asiloidflies.si.edu/specimen-map-Apioceridae?genus=Apiocera

http://asiloidflies.si.edu/specimen-table-Apioceridae?genus=Apiocera

Apiocera Westwood 1835: 448

##### Distribution

Argentina, Australia, Canada, Chile, Mexico, South Africa, USA

#### Ripidosyrma

Hermann, 1909

urn:lsid:zoobank.org:act:7822B44A-767F-4B67-9BE3-19F53894CABB

http://biodiversitylibrary.org/page/33103604

http://dx.doi.org/10.5281/zenodo.30975

http://biostor.org/reference/101352

http://treatment.plazi.org/id/D74EC1E3-A0DD-6EB1-6269-4F121D8FAC36

http://www.morphbank.net/Browse/ByImage/?tsn=999022154

http://asiloidflies.si.edu/specimen-map-apioceridae?genus=Apiocera&subgenus=Ripidosyrma

http://asiloidflies.si.edu/specimen-table-apioceridae?genus=Apiocera&subgenus=Ripidosyrma

Ripidosyrma Hermann 1909: 104
Apiocera , new junior synonymy by Paramonov 1950 : 103
Apiocera (Ripidosyrma) , new subgeneric rank by Yeates and Irwin 1996 : 288

##### Distribution

South Africa

#### Apiocera (Ripidosyrma) alastor

(Walker, 1849)

urn:lsid:zoobank.org:act:C4D38C42-45E5-48A0-B55A-29BC06FFB72A

http://biodiversitylibrary.org/page/38706155

http://dx.doi.org/10.5281/zenodo.30972

http://www.biodiversitylibrary.org/item/119308

http://www.gbif.org/species/1611657

http://treatment.plazi.org/id/03EF3D32-FFB6-5C2C-FFF5-FAD25186B0F7

http://plazi.cs.umb.edu/GgServer/search?taxonomicName.isNomenclature=true&taxonomicName.exactMatch=true&taxonomicName.order=Diptera&taxonomicName.species=alastor

http://www.morphbank.net/Browse/ByImage/?tsn=999022155

http://eol.org/pages/718862/overview

http://asiloidflies.si.edu/specimen-map-Apioceridae?genus=Apiocera&species=alastor

http://asiloidflies.si.edu/specimen-table-Apioceridae?genus=Apiocera&species=alastor

Asilus alastor Walker 1849: 444
Apiocera (Ripidosyrma) alastor , new combination by Stuckenberg 1968 : 124
Apiocera (Ripidosyrma) africana Paramonov, 1950, new junior synonymy by Yeates 1994 : 124
Apiocera (Ripidosyrma) alastor ZooBank GUID B745D2C2-8CB6-4C95-970E-B3C3246B7A73 Plazi original treatment GUID B745D2C2-8CB6-4C95-970E-B3C3246B7A73

##### Distribution

South Africa (Northern Cape, Western Cape)

##### Notes

Institutions with specimens: BMNH, SAMC, ZSMC.

#### Apiocera (Ripidosyrma) badipeniculata

Yeates, 1994

urn:lsid:zoobank.org:act:0335A2B2-8650-419C-A645-7FD4A441B690

http://dx.doi.org/10.5281/zenodo.15791

http://hdl.handle.net/10520/AJA03040798_231

http://treatment.plazi.org/id/0335A2B2-8650-419C-A645-7FD4A441B690

http://plazi.cs.umb.edu/GgServer/search?taxonomicName.isNomenclature=true&taxonomicName.exactMatch=true&taxonomicName.genus=Apiocera&taxonomicName.species=badipeniculata

http://www.gbif.org/species/1611753

http://eol.org/pages/424464/overview

http://asiloidflies.si.edu/specimen-map-Apioceridae?genus=Apiocera&species=badipeniculata

http://asiloidflies.si.edu/specimen-table-Apioceridae?genus=Apiocera&species=badipeniculata

Apiocera badipeniculata Yeates 1994: 128

##### Distribution

South Africa (Northern Cape/Western Cape). The type locality of Tankwa Karoo, which can be interpreted to overlap with the Tankwa Karoo National Park, straddles the border of the Northern and Western Cape provinces.

##### Notes

Institutions with specimens: SAMC.

#### Apiocera (Ripidosyrma) braunsi

Melander, 1907

urn:lsid:zoobank.org:act:5C0A5C13-EC67-428B-9712-4734564EA06D

http://biodiversitylibrary.org/page/47141510

http://dx.doi.org/10.5281/zenodo.16194

http://biodiversitylibrary.org/page/47141509

http://treatment.plazi.org/id/5C0A5C13-EC67-428B-9712-4734564EA06D

http://plazi.cs.umb.edu/GgServer/search?taxonomicName.isNomenclature=true&taxonomicName.exactMatch=true&taxonomicName.genus=Apiocera&taxonomicName.species=braunsi

http://www.gbif.org/species/1611644

http://www.morphbank.net/Browse/ByImage/?tsn=999022156

http://eol.org/pages/718861/overview

http://asiloidflies.si.edu/specimen-map-Apioceridae?genus=Apiocera&species=braunsi

http://asiloidflies.si.edu/specimen-table-Apioceridae?genus=Apiocera&species=braunsi

Apiocera braunsi Melander 1907: 126

##### Distribution

South Africa (Eastern Cape, Western Cape)

##### Notes

Institutions with specimens: AMGS, BMNH, NMSA, SAMC, SANC, SMNS, TMSA, USNM, ZSMC.

Two museum collections claim to house the holotype of *A. braunsi*. Stuckenberg (1968), in transferring *Asilus alastor* to *Apiocera*, notes that he has seen "part of the type series of *braunsi*", but he does not indicate where these specimens are deposited. However, Melander (1907), in describing *A. braunsi*, states that he has only seen a single specimen, a male. What appears to have happened is that J. Brauns collected three specimens on 1 January 1905 of an *Apiocera* species and dispersed them shortly thereafter to two individuals. One specimen was sent along with other Asilidae and Mydidae to A.L. Melander in the USA and two specimens ended up in the Transvaal Museum (TMSA, now Ditsong National Museum of Natural History) in Pretoria where Brian Stuckenberg must have studied them. The entire Transvaal Museum Diptera collection was donated to the KwaZulu-Natal Museum (NMSA) in 1974 (Londt 1998) and this is how a male and a female of *A. braunsi* arrived at the NMSA where the male was regarded as the holotype (Yeates 1994). Yeates (1994) mentions that the A.L. Melander collection was acquired by the Smithsonian Institution's National Museum of Natural History (USNM) and that no specimen matching the collection event data could be found. However, there is a specimen clearly marked as holotype in the USNM collection that arrived there in 1961 when the Melander collection was incorporated. NMSA Diptera curator Burgert Muller and I compared images of the putative male holotype specimens in each collection and the USNM specimen does match the whole habitus illustration provided in Melander (1907) perfectly (the way the abdomen is bent dorsally and the shape of the epandrial plume, compare lateral view of the holotype in original publication with image of USNM holotype). Therefore, we regard the USNM specimen to be the holotype and the NMSA specimens (1 male and 1 female) as non-type specimens.

## Discussion

The present cybercatalog summarizes taxonomic information on Afrotropical Apioceridae by utilizing freely available digital resources from numerous data repositories. While the initial work to locate information in or upload images, treatments, and publications to data repositories might seem daunting, the long-term effects of using openly accessible digital content through persistent identifiers and linked data far outweighs the time spent. Open-access science, in which taxonomists share data freely and electronically, will on the one hand advance the discipline within the biological sciences and on the other hand make taxonomic hypotheses easily testable through the re-use and re-purpose of previously gathered data with the addition of new data. A taxonomist's dream of having access to all previously published descriptions, images, illustrations, and notes of a particular taxon can become true if the community supports sharing research results in machine-readable form and structured data repositories as exemplified above.

At the same time, this approach is opening up taxonomy to the widest possible community and only this step allows to position taxonomy as a central resource within the life sciences, applied fields of study, and beyond to the public and society at large. We taxonomists have always claimed to fulfill this role, but never understood why it did not happen.

## Supplementary Material

XML Treatment for Apiocera

XML Treatment for Ripidosyrma

XML Treatment for Apiocera (Ripidosyrma) alastor

XML Treatment for Apiocera (Ripidosyrma) badipeniculata

XML Treatment for Apiocera (Ripidosyrma) braunsi

## Figures and Tables

**Figure 1a.**
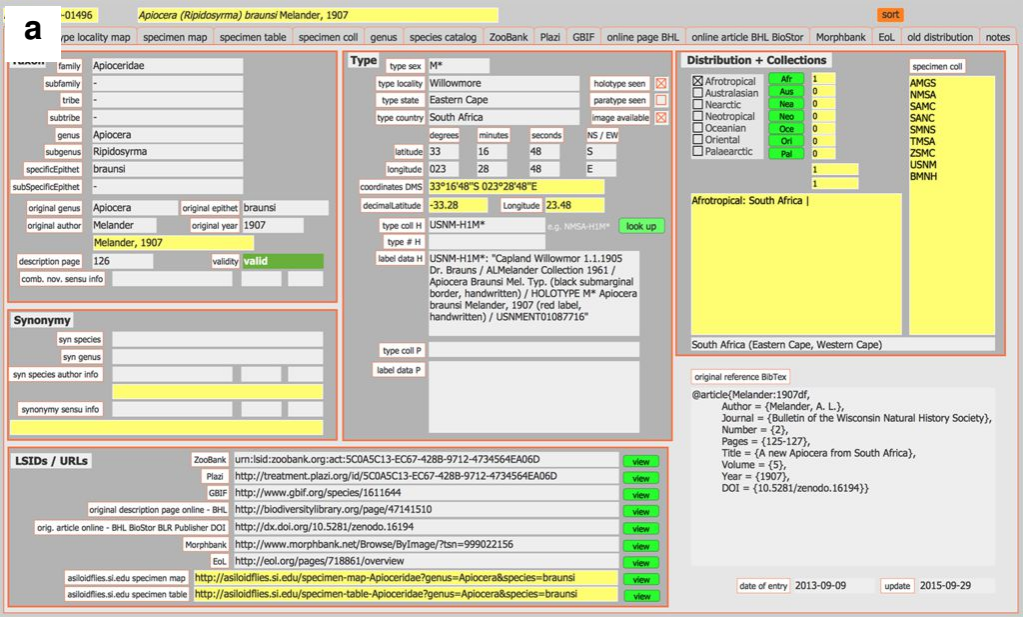
Species tab.

**Figure 1b.**
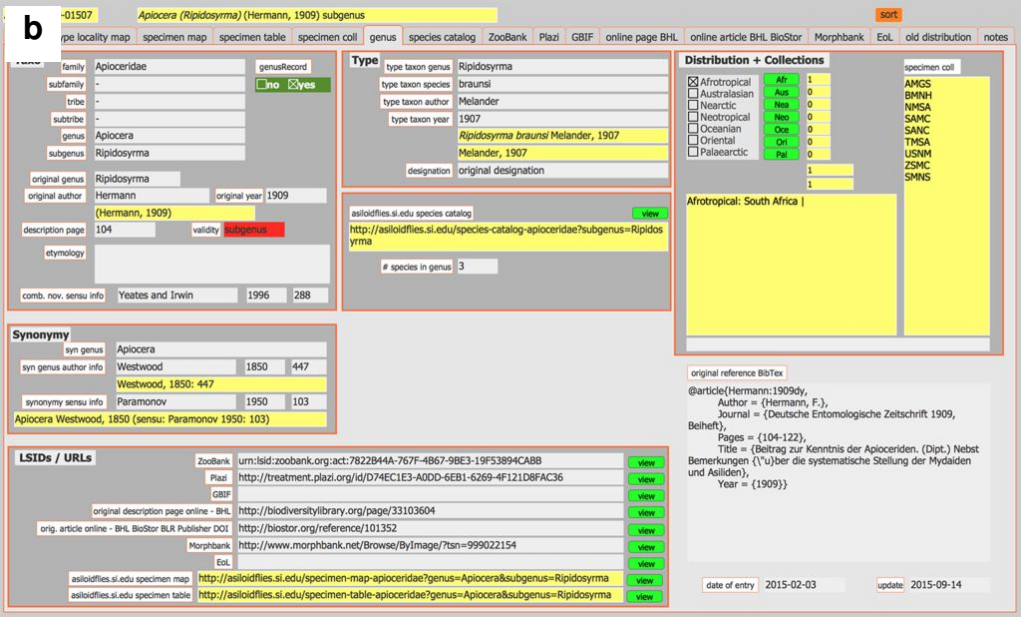
Genus tab.
